# The bacteriophage-encoded regulator PemR attenuates *Pseudomonas aeruginosa* virulence by hijacking quorum sensing and metabolism

**DOI:** 10.1128/jvi.02071-25

**Published:** 2026-02-05

**Authors:** Wenbo Yan, Yingxin Yang, Jiahui Cao, Mengyao Zhang, Yiming Yang, Chao Li, Nan Zhang, Lixin Yuan, Meng Li, Lijun Liu, Yani Zhang, Shiwei Wang, Tietao Wang

**Affiliations:** 1 Key Laboratory of Resources Biology and Biotechnology in Western China, Ministry of Education, College of Life Sciences, Northwest University 4268, Xi’an, Shaanxi, China; 2 School of Basic Medical Science, Xi’an Medical University, Xi’an, Shaanxi, China; Michigan State University, East Lansing, Michigan, USA

**Keywords:** bacteriophage, *Pseudomonas aeruginosa*, quorum-sensing system, virulence regulation, anthranilate metabolism

## Abstract

The global challenge of multidrug-resistant *Pseudomonas aeruginosa* demands innovative anti-virulence approaches. We characterize PemR, a novel transcriptional regulator encoded by *P. aeruginosa* phage PAYQ66, which orchestrates multimodal virulence attenuation in *P. aeruginosa*. Biochemical analyses demonstrated that PemR directly binds to the *mvfR* promoter, resulting in significant repression of this key quorum-sensing regulator. This repression, in turn, induces profound metabolic reprogramming by redirecting metabolic flux away from PQS biosynthesis toward catechol accumulation. PemR globally attenuates virulence phenotypes, including pyocyanin production, rhamnolipid synthesis, motility, and biofilm formation. Transcriptomic profiling further reveals that PemR upregulates *rsmA* to suppress the type VI secretion system, thereby potentially modulating the host’s interaction with competing bacteria in polymicrobial environments. The motility analysis shows that PemR suppresses bacterial swimming, swarming, and twitching at gene expression levels. Overall, infection models demonstrate that PemR significantly inhibits bacterial virulence. As the first reported phage-encoded transcriptional hijacker of the *mvfR* regulon, PemR concurrently disrupts quorum sensing and bacterial pathogenicity, offering a strategic blueprint for novel anti-virulence therapeutics targeting multidrug-resistant pathogens.

**IMPORTANCE:**

This study identifies a novel bacteriophage-encoded regulator PemR that simultaneously disrupts multiple virulence pathways in the opportunistic pathogen *P. aeruginosa*. By hijacking a key bacterial quorum-sensing (QS) system and reprogramming host metabolism, PemR significantly reduces pathogenicity without killing the bacteria. This work reveals a sophisticated strategy phages use to manipulate their hosts and provides a promising blueprint for developing next-generation anti-virulence therapeutics. Such approaches aim to disarm dangerous bacteria rather than eliminate them, potentially slowing the emergence of antibiotic resistance and offering new strategies against multidrug-resistant infections.

## INTRODUCTION

Bacterial infections pose a grave threat to global public health (1). While antibiotics have served as the cornerstone of treatment since their discovery, widespread misuse has accelerated the evolution of drug resistance in pathogenic bacteria (2). This alarming trend has transformed antimicrobial resistance into a pressing medical challenge, with projections indicating that drug-resistant infections may claim over 10 million lives annually by 2050 (1, 3). Ranked among the top three opportunistic pathogens, *P. aeruginosa* demonstrates remarkable propensity for developing multidrug resistance (MDR) under antibiotic selection pressure. MDR strains significantly compromise clinical outcomes by limiting therapeutic options and elevating mortality rates, prompting the World Health Organization to designate it as a critical priority pathogen requiring novel treatment strategies (3, 4).

One emerging strategy is anti-virulence therapy, which disrupts pathogenic mechanisms without affecting bacterial growth or viability (5). The quorum sensing (QS) system in *P. aeruginosa* serves as a master regulatory network that orchestrates multiple virulence-associated phenotypes, including (but not limited to) (i) flagellar and pilus-mediated motility, (ii) efflux pump expression and activity, (iii) multi-stage biofilm formation, and (iv) environmental adaptation strategies (6–9). This sophisticated cell-to-cell communication system fundamentally governs the spatiotemporal expression of virulence determinants during infection (10). *P. aeruginosa* employs three hierarchically organized QS systems: the Las, Rhl, and *Pseudomonas* quinolone signal (PQS) systems (11, 12). The Las system, comprising the transcriptional regulator LasR and the autoinducer synthase LasI, serves as the top-tier regulatory module. LasI catalyzes the biosynthesis of N-(3-oxododecanoyl)-L-homoserine lactone (3-OC_12_-HSL), which upon binding to LasR forms an active complex that modulates the expression of key virulence factors including elastase (LasB) and exotoxin A (ToxA) (13, 14). The Rhl system, composed of the transcriptional regulator RhlR and autoinducer synthase RhlI, constitutes the second-tier regulatory module in *P. aeruginosa*. RhlI catalyzes the production of N-butyryl-L-homoserine lactone (C_4_-HSL), which upon binding to RhlR activates the expression of biosynthetic gene clusters responsible for rhamnolipid production, a crucial virulence determinant facilitating biofilm maturation and immune evasion (12, 15, 16). The PQS system represents the third regulatory tier, which is centered on the transcriptional regulator MvfR (also designated PqsR) (17). As the master controller of the *pqs* operon, MvfR mediates the enzymatic conversion of anthranilate into 2-heptyl-4-quinolone (HHQ), which is subsequently modified to produce the namesake PQS signal molecule (2-heptyl-3-hydroxy-4-quinolone) (18). This signaling cascade ultimately governs the production of key virulence determinants, including pyocyanin and mature biofilm architectures (19). Therefore, targeting the QS system provides a promising direction for developing novel therapies that can effectively control *P. aeruginosa* pathogenicity by interfering with the QS system (20).

Bacteriophages are viruses that can specifically infect bacteria without affecting eukaryotic cellular processes (21). They attach to susceptible bacterial cells and inject their nucleic acids into the host. Ultimately, new phage particles assemble within the bacterial cell and are released through cell lysis (22). Given the emergence of multidrug-resistant bacteria, phages are receiving increasing attention as antimicrobial agents (23). For billions of years, bacteria and bacteriophages have maintained a dynamic equilibrium through an ongoing evolutionary arms race, characterized by cyclical adaptations and counter-adaptations (24). To enhance their lytic efficiency, bacteriophages have acquired an arsenal of specialized proteins, including endolysins and nucleases, which target critical bacterial functions (25, 26). Conversely, bacteria have evolved sophisticated defense strategies, exemplified by CRISPR-Cas systems and restriction-modification (R-M) systems, to resist phage predation and ensure their survival (27). The QS system, a crucial regulatory mechanism in bacteria, plays an active role in anti-phage defense. Evidence indicates that *P. aeruginosa* employs QS-mediated strategies to counteract phage infection (28, 29). Notably, a recent study demonstrated that PQS, produced in response to phage infection, can repel healthy *P. aeruginosa* populations on solid agar plates, effectively containing phage propagation (30). In response to QS-based bacterial defenses, bacteriophages have evolved specialized anti-QS effector proteins to subvert host signaling. *P. aeruginosa* DMS3 phage-encoded Aqs1 and LMA2 phage protein PIT2 directly interact with the LasR transcriptional regulator, effectively hijacking the QS regulatory network in their bacterial hosts (31, 32). Meanwhile, the phage vB_Pae_QDWS-encoded Gp21 protein protects *P. aeruginosa* against phage infection by interfering with QS and reducing type IV pilus synthesis, thereby limiting phage adsorption (33). Consequently, the identification and mechanistic characterization of anti-QS proteins from bacteriophages may provide a novel theoretical framework for developing next-generation antimicrobial agents that attenuate bacterial virulence and pathogenicity (34).

This study reports a novel transcriptional regulator PemR (Phage-encoded MvfR transcriptional regulator), derived from the *P. aeruginosa* lytic phage PAYQ66. PemR inhibits the key QS system regulator MvfR, reprogramming host metabolism toward catechol accumulation and significantly attenuating virulence phenotypes, including pyocyanin production, motility, biofilm formation, and rhamnolipid synthesis. Furthermore, PemR upregulates *rsmA* to suppress the type VI secretion system (T6SS), thereby reducing the environmental competitiveness of the host bacterium. Through systematic phage gene regulation screening, this study successfully identified and characterized novel QS system inhibitory proteins that effectively attenuate *P. aeruginosa* virulence and pathogenicity. Mechanistic investigations revealed that the transcriptional regulator binds to the *mvfR* promoter to repress its expression, thereby suppressing virulence factor production and biofilm formation. The findings not only advance our understanding of phage-host interactions but also provide a molecular framework for developing innovative anti-virulence therapeutics targeting bacterial QS systems.

## RESULTS

### The phage-encoded transcriptional regulator PemR suppresses *mvfR* expression via direct promoter binding

**Fig 1:**
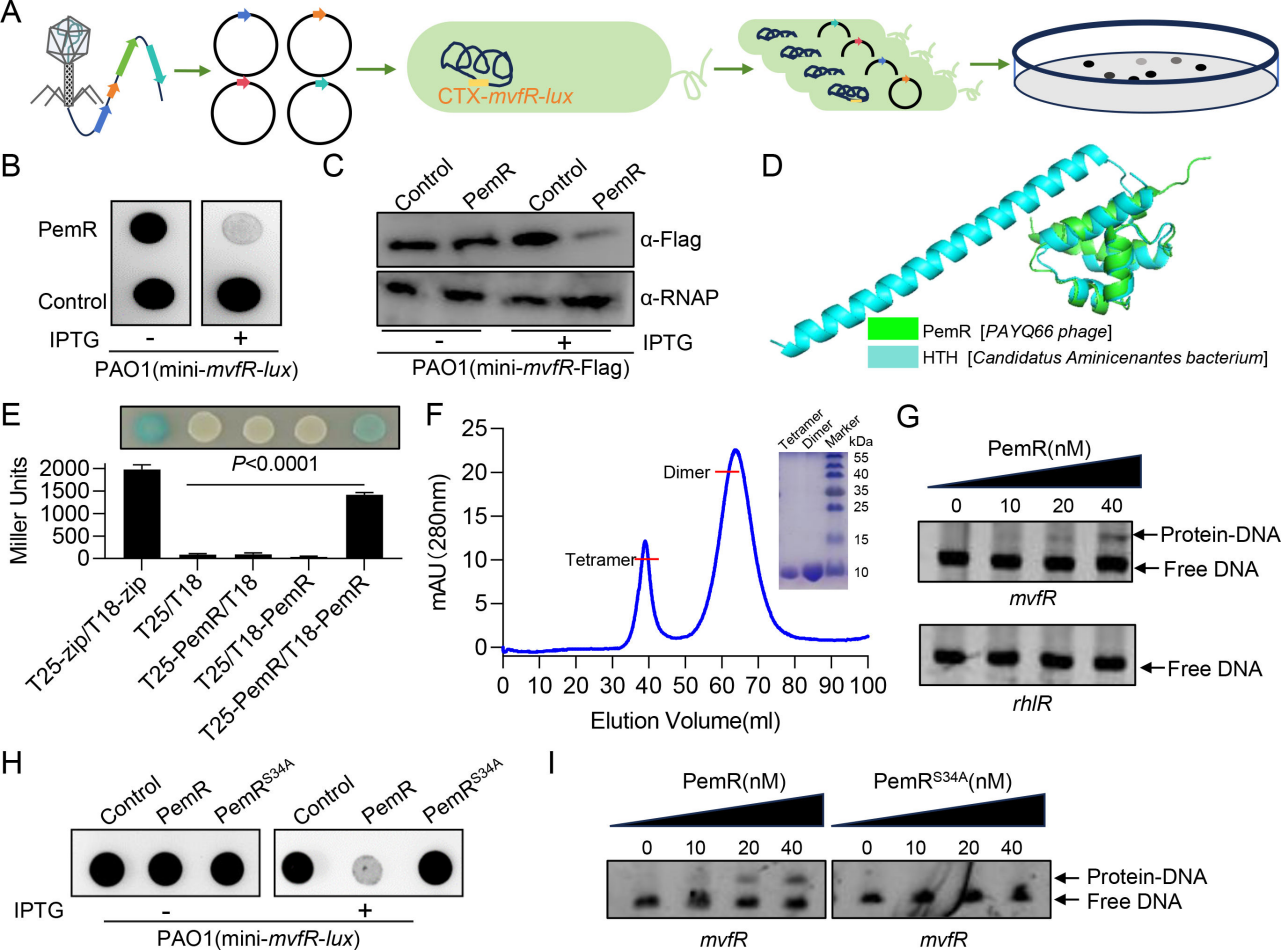
The phage-encoded transcriptional regulator PemR suppresses *mvfR* expression by directly binding to its promoter. (**A**) Schematic diagram of the experimental procedure for identifying phage genes that regulate the expression of *mvfR* in *P. aeruginosa*. Early-expressed phage genes were individually cloned into the inducible plasmid pME6032 and transformed into PAO1 (integrated with mini-*mvfR-lux*) strains. Subsequently, the luminescence of each constructed strain was examined after growth at 37°C for 12 h on LB plates with 0.5 mM IPTG. (**B**) Overnight cultures of *P. aeruginosa* (mini-*mvfR-lux*) harboring either pME6032 (Control) and pME6032-*pemR* (PemR) were spotted onto LB plates with or without 0.5 mM IPTG and incubated at 37°C. Luminescence was imaged after 12 h. (**C**) *P. aeruginosa* strains (integrated with mini-*mvfR*-Flag) harboring either the empty vector pME6032 (Control) or pME6032-*pemR* (PemR) were cultured to an OD_600_ of 1.0. Equal amounts of samples were loaded onto SDS-PAGE gels and detected using anti-Flag antibody. α-RNA polymerase (RNAP) served as the loading control. (**D**) Superposition of the 3D structure of the helix-turn-helix (HTH) transcriptional regulator from *Candidatus aminicenantes bacterium* (Sequence ID: UCE40751.1) and PemR structures using Alpha Fold3 and visualized by PyMOL. The structures are colored green and cyan, respectively. (**E**) Qualitative and quantitative analysis of BACTH assay. Protein-protein interaction between PemR and PemR was assessed using bacterial adenylate cyclase-based two-hybrid (BACTH) system coupled with β-galactosidase assay. *E. coli* BTH101 strains expressing pKT25-zip/pUT18C-zip plasmids served as positive controls, while those harboring empty pKT25/pUT18C, pKT25-PemR/pUT18C, and pKT25/pUT18C-PemR vectors were used as negative controls. (**F**) SEC analysis of PemR. SEC chromatographic profiles of the purified PemR in 10 mM Tris-HCl, 500 mM NaCl buffer with Seplife S-100. The inset shows protein analysis of the indicated peaks by SDS-PAGE and Coomassie Brilliant Blue-R250 staining. (**G**) EMSA reveals that PemR binds to and shifts the *mvfR* promoter but not the *rhlR* promoter. Each reaction mixture contained 2.0 ng/μL of *mvfR* and *rhlR* PCR products. Protein concentrations are indicated above the lanes. (**H**) Overnight cultures of *P. aeruginosa* (mini-*mvfR-lux*) harboring either pME6032 (Control), pME6032-*pemR* (PemR), and pME6032-*pemR^S34A^* (PemR^S34A^) were spotted onto LB plates with or without 0.5 mM IPTG and incubated at 37°C. Luminescence was imaged after 12 h. (**I**) EMSA reveals that PemR binds to and shifts the *mvfR* promoter, whereas PemR^S34A^ does not bind to the *mvfR* promoter. Each reaction mixture contained 2.0 ng/μL of *mvfR* PCR products. Protein concentrations are indicated above the lanes. (**B, C, and E–I**) Data are representative of three independent replicates. (**E**) Data shown are representative from three independent experiments. Error bars indicate mean ± standard deviation. Statistical significance was calculated using paired *t*-test.

The *P. aeruginosa* lytic phage PAYQ66 was isolated from a wastewater reservoir at Northwest University in Xi'an, Shaanxi Province, China. It features a linear double-stranded DNA genome of 48,146 bp with a GC content of 55%. The genome is predicted to encode 64 open reading frames (ORFs) and one tRNA gene (Table S1). Morphologically, PAYQ66 belongs to the *Siphoviridae* family, characterized by an isometric head approximately 60 nm in diameter and a long, non-contractile tail measuring about 190 nm in length (Fig. S1). We aimed to identify phage genes from PAYQ66 that affect *mvfR* expression, since *mvfR* is a key LysR-type transcriptional regulator controlling PQS synthesis (18). Therefore, we cloned 34 hypothetical ORFs of phage PAYQ66 into the high-expression plasmid pME6032 for functional screening (Table S2. The 34 ORFs were transformed into *P. aeruginosa* carrying mini-*mvfR-lux*, then plated on LB agar containing IPTG to screen for strains with altered luminescence (Fig. 1A). PemR (PAYQ66_*orf*47) was found to significantly inhibit *mvfR* expression, and RT-qPCR analysis confirmed that *pemR* is an early-expressed gene compared with the capsid protein gene *orf18* (Fig. 1B; Fig. S2). To further validate the screening results, we dynamically monitored the effect of PemR on *mvfR* expression. Throughout bacterial growth, PemR consistently and significantly suppressed *mvfR* expression (Fig. S3). Western blot analysis using whole-cell lysates of PemR-expressing PAO1 further confirmed that PemR significantly suppresses intracellular protein levels of *mvfR* (MvfR-Flag), providing additional validation of its inhibitory effect on *mvfR* expression (Fig. 1C).

To investigate how PemR suppresses *mvfR* expression, we performed BLAST and structural prediction analyses. The results revealed that PemR shares 41.67% identity with a helix-turn-helix (HTH) transcriptional regulator from *Candidatus Aminicenantes* bacterium (Sequence ID: UCE40751.1), and AlphaFold3 predictions demonstrated high structural similarity between them (PemR: pTM = 0.88; HTH: pTM = 0.74) (Fig. 1D; Fig. S4A and B) (10). The structural model, also generated by AlphaFold3, further supports the formation of a PemR dimer (Fig. S4C). These findings suggest that PemR may function as a transcriptional regulator. As a hallmark feature of HTH-containing proteins, dimerization is essential for DNA recognition. We confirmed PemR’s self-association through (i) bacterial two-hybrid interaction assays and (ii) identification of dimeric/tetrameric species by analytical size-exclusion chromatography (SEC). The results indicate that PemR interacts with itself to form multimers (Fig. 1E). SEC revealed two peaks for PemR (7.8 kDa), suggesting the formation of two distinct multimeric forms. The elution volume of the first peak was similar to that of HaloTag (34 kDa), indicating that the first peak corresponds to a tetramer. The elution volume of the second peak is similar to that of RNase A (15 kDa), suggesting that this peak corresponds to a dimer. (Fig. 1F; Fig. S5) (35). This oligomerization capacity, combined with its predicted HTH domain, strongly supports PemR’s role as a transcriptional regulator of *mvfR*. EMSA assays revealed specific complex formation between PemR and the *mvfR* promoter region, providing direct mechanistic evidence for PemR-mediated transcriptional repression (Fig. 1G). To substantiate PemR-mediated repression of *mvfR* via promoter binding, we identified the serine^34^ as a conserved residue within its HTH domain through multiple sequence alignment (Fig. S6A). Site-directed mutagenesis of serine to alanine (PemR^S34A^) abolished the repression of *mvfR*, with its expression level remaining unchanged (Fig. 1H; Fig. S6B and C). Furthermore, EMSA assays with the purified PemR^S34A^ protein confirmed the loss of PemR^S34A^ binding to the *mvfR* promoter (Fig. 1I; Fig. S6D). Together, these results demonstrate that PemR functions as a transcriptional repressor that directly binds to the *mvfR* promoter and suppresses its expression.

### PemR suppresses PQS and enhances catechol production via *mvfR*

**Fig 2:**
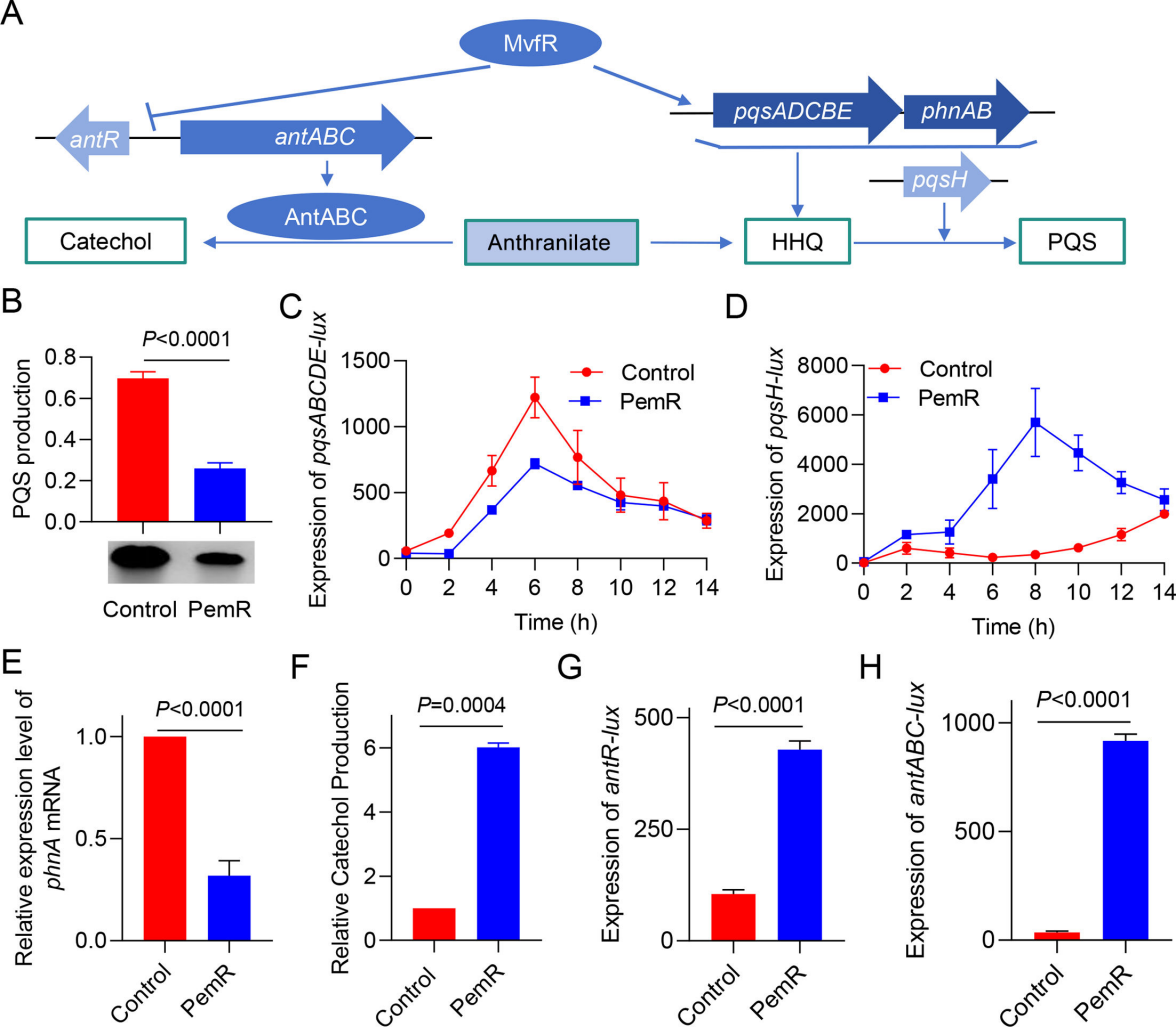
PemR modulates *mvfR* to suppress PQS and enhance catechol production. (**A**) Schematic illustration of *mvfR*-mediated regulation of anthranilate metabolism. Anthranilate, the precursor of PQS signaling molecules, can be catabolized into catechol via the AntABC metabolic pathway. (**B**) Thin-layer chromatography (TLC) analysis of PQS production in *Pseudomonas aeruginosa* strains harboring either the empty vector pME6032 (Control) or pME6032-*pemR* (PemR). The intensity of bands was quantified using Fiji ImageJ. (**C–D**) PemR inhibits *pqsH* and *pqsABCDE* expression. The transcriptional activity of *pqsH* and *pqsABCDE* promoter was analyzed in PAO1 harboring pME6032 (Control) and PAO1 harboring *pemR* (PemR) using reporter gene assay. (**E**) PemR downregulates *phnA* expression. Relative mRNA levels of *phnA* were quantified by qRT-PCR in PAO1 (Control) versus PAO1 expressing *pemR* (PemR). (**F**) PemR enhances catechol production. Culture supernatants from PAO1 (Control) and PAO1 expressing *pemR* (PemR) were analyzed by HPLC, with relative quantification of components performed using LabSolutions software based on peak areas. (**G–H**) PemR upregulates *antABC* and *antR* expression. Promoter activities of *antABC* and *antR* were measured in PAO1 (Control) versus PAO1 expressing *pemR* (PemR). (**B–H**) Data are representative of two (**F**) or three (**B, C, D, E, H**) independent experiments. Error bars indicate mean ± standard deviation. Statistical significance was calculated using paired *t*-test.

In *P. aeruginosa*, anthranilate serves as the precursor for PQS biosynthesis (10). The PQS synthesis pathway involves the enzymatic actions of PqsABCDE, PhnAB, and PqsH, with the entire metabolic cascade being positively regulated by the transcriptional regulator MvfR (19, 36). Conversely, MvfR negatively regulates and suppresses the expression of *antABC*, thereby inhibiting catechol production (Fig. 2A). Given PemR’s repression of *mvfR* (an activator of PQS synthesis and a repressor of *antABC*), we predicted that PemR-expressing strains would shift anthranilate flux from PQS to catechol (36, 37).

To test this prediction, we compared PQS levels between wild-type PAO1 (Control) and isogenic PemR-expressing PAO1 (PemR) using TLC analysis. Densitometric quantification revealed a 2.7-fold (*P* < 0.0001) reduction in PQS production in PemR-expressing PAO1 (PemR) relative to wild-type PAO1 (Control) (Fig. 2B). Furthermore, we quantified the expression levels of the PQS biosynthetic cluster genes (*pqsABCDE* and *pqsH* by luciferase reporter assay, *phnA* by RT-qPCR) in PemR-expressing strains, which revealed significantly reduced transcript levels compared to the control strain (Fig. 2C through E). The metabolic shift caused by PQS suppression resulted in significant catechol accumulation. Quantitative HPLC measurements demonstrated a 6-fold increase (*P =* 0.0004) in catechol concentration. Concurrently, significant upregulation was observed for both the catechol biosynthesis gene cluster *antABC* and its transcriptional regulator *antR* in PemR-expressing PAO1 (PemR) versus wild-type PAO1 (Control) (Fig. 2F through H). These findings indicate that the PemR-mediated repression of *mvfR* redirects anthranilate metabolic flux from PQS biosynthesis toward catechol accumulation, thereby altering the metabolic landscape of *P. aeruginosa*.

### PemR attenuates *P. aeruginosa* virulence

**Fig 3:**
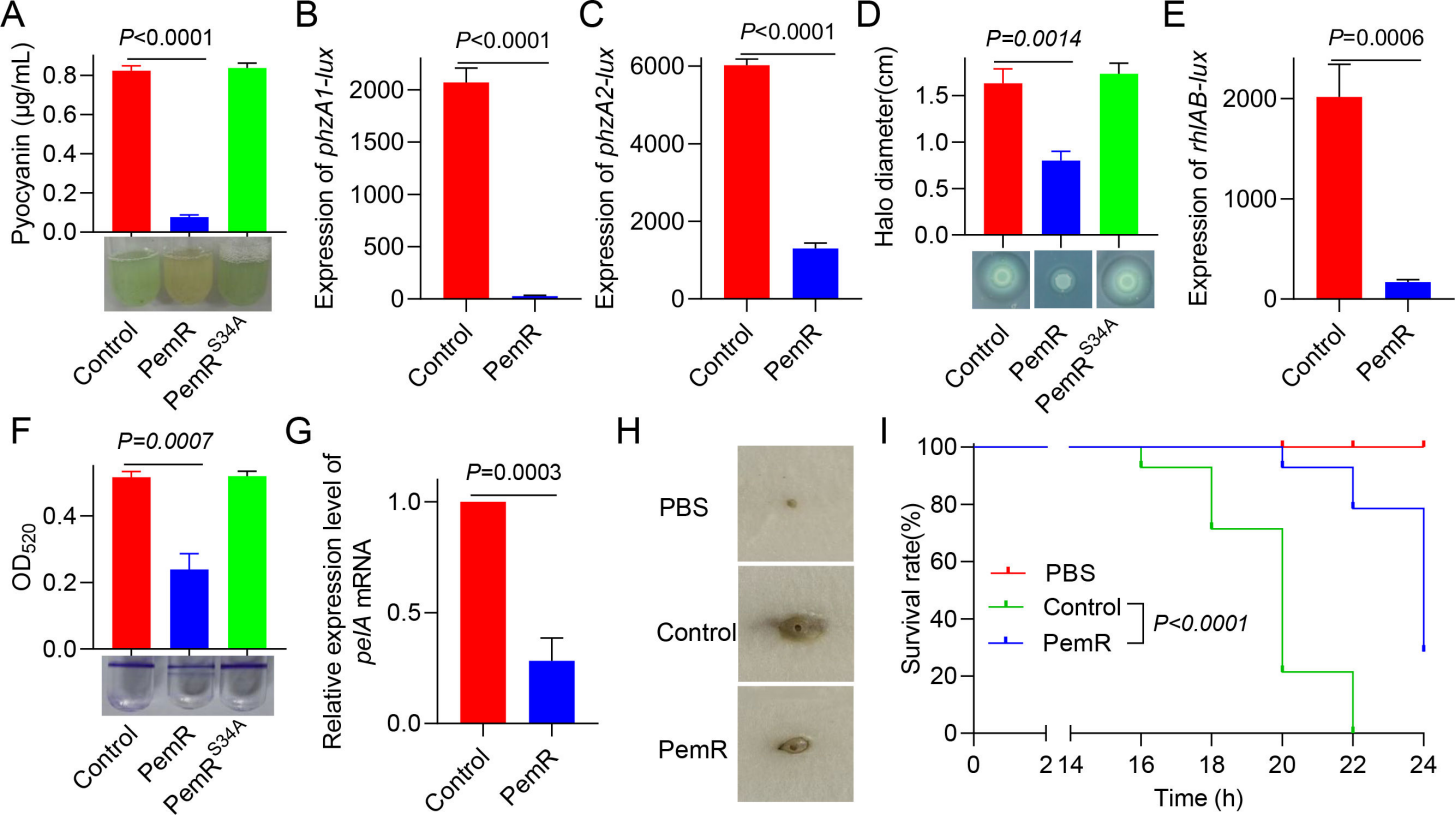
PemR attenuates *P. aeruginosa* virulence. (**A**) Pyocyanin production was measured in PAO1 (Control), PAO1(PemR), and PAO1(PemR^S34A^) strains following 12 h of growth in LB medium. (**B–C**) PemR suppresses *phzA1* and *phzA2* expression. Promoter activities of *phzA1* and *phzA2* were measured in PAO1 (Control) versus PAO1 expressing *pemR* (PemR). (**D**) Rhamnolipid production by PAO1 (Control), PAO1(PemR), and PAO1(PemR^S34A^) strains on CTAB-methylene blue plates. (**E**) PemR suppresses *rhlAB* expression. Promoter activity of *rhlAB* was measured in PAO1 (Control) versus PAO1 expressing *pemR* (PemR). (**F**) Biofilm formation in PAO1 (Control), PAO1(PemR), and PAO1(PemR^S34A^) strains was assessed by crystal violet staining (lower panel) and optical density measurement (upper panel). (**G**) PemR downregulates *pelA* expression. Relative mRNA levels of *pelA* were quantified by qRT-PCR in PAO1 (Control) versus PAO1 expressing pemR (PemR). (**H–I**) Pathogenicity assessment of PAO1 (Control) versus PAO1 expressing *pemR* (PemR) using Chinese cabbage (*Brassica rapa*) and greater wax moth (*Galleria mellonella*) infection models. PBS served as the negative control. (**A, D, F, and H**) Data shown are representative from three independent experiments. (**A–G**) Data are representative of three independent replicates. Error bars indicate mean ± standard deviation. Statistical significance was calculated using paired *t*-test. (**I**) Statistical significance was calculated using Log-rank (Mantel–Cox) test.

MvfR, as a key regulator of the QS system, coordinates the expression of multiple *P. aeruginosa* virulence factors, including pyocyanin, protease, biofilm formation, and rhamnolipid production (18). Therefore, we assessed the impact of PemR on these virulence factors in PAO1. Quantitative analyses revealed that PemR-expressing PAO1 (PemR) exhibited a 90% reduction in pyocyanin (*P* < 0.0001), 50% decrease in rhamnolipids (*P* = 0.0014), and a 2.16-fold impairment in biofilm formation (*P* = 0.007), but showed no significant change in protease production (*P* = 0.3739) compared to wild-type PAO1 (Control) (Fig. 3A, D and F; Fig. S7A). We further examined the expression levels of key virulence-associated gene clusters in PemR-expressing strains (PemR), including the pyocyanin biosynthesis genes (*phzA1* and *phzA2*), rhamnolipid synthesis operon (*rhlAB*), and biofilm matrix components (*cdrA* protein and polysaccharide synthesis gene *pelA*) (Fig. 3B, C, E and G; Fig. S7B). The results demonstrated that PemR significantly suppressed their expression compared to the control strain. In contrast, no significant difference in virulence phenotypes was observed between the PemR^S34A^-expressing strains (PemR^S34A^) and the control strain. The QS network’s core regulators (RhlI/R, LasI/R, and MvfR) exhibit reciprocal control: RhlR inhibits *mvfR*, whereas MvfR stimulates *rhlR*. This cross-regulation explains the observed RhlI-R significantly suppressed in PemR-expressing strains, demonstrating system-wide QS disruption (Fig. S7C and D) (18).

Concurrently, pathogenicity was assessed using both plant and insect infection models. The Chinese cabbage infection model was used as a plant-based system to assess *P. aeruginosa* pathogenicity, where tissue maceration (rotting) serves as a readout for bacterial virulence (38, 39). In this model, PemR-expressing PAO1 caused a significantly reduced rotten area compared to the wild-type control (Fig. 3H). Similarly, in the *Galleria mellonella* infection model, PemR expression resulted in 78% larval survival, in stark contrast to the complete lethality (0% survival) caused by wild-type PAO1 at 22 h (*P* < 0.0001) (Fig. 3I). Collectively, these results confirm that PemR significantly attenuates *P. aeruginosa* virulence in both plant and insect hosts. These findings demonstrate that PemR significantly attenuates key virulence phenotypes, including pyocyanin production, rhamnolipid synthesis, and biofilm formation, and reduces pathogenicity in both plant and insect infection models, highlighting its broad anti-virulence potential.

### Global transcriptome profiling of *P. aeruginosa* upon PemR expression

**Fig 4:**
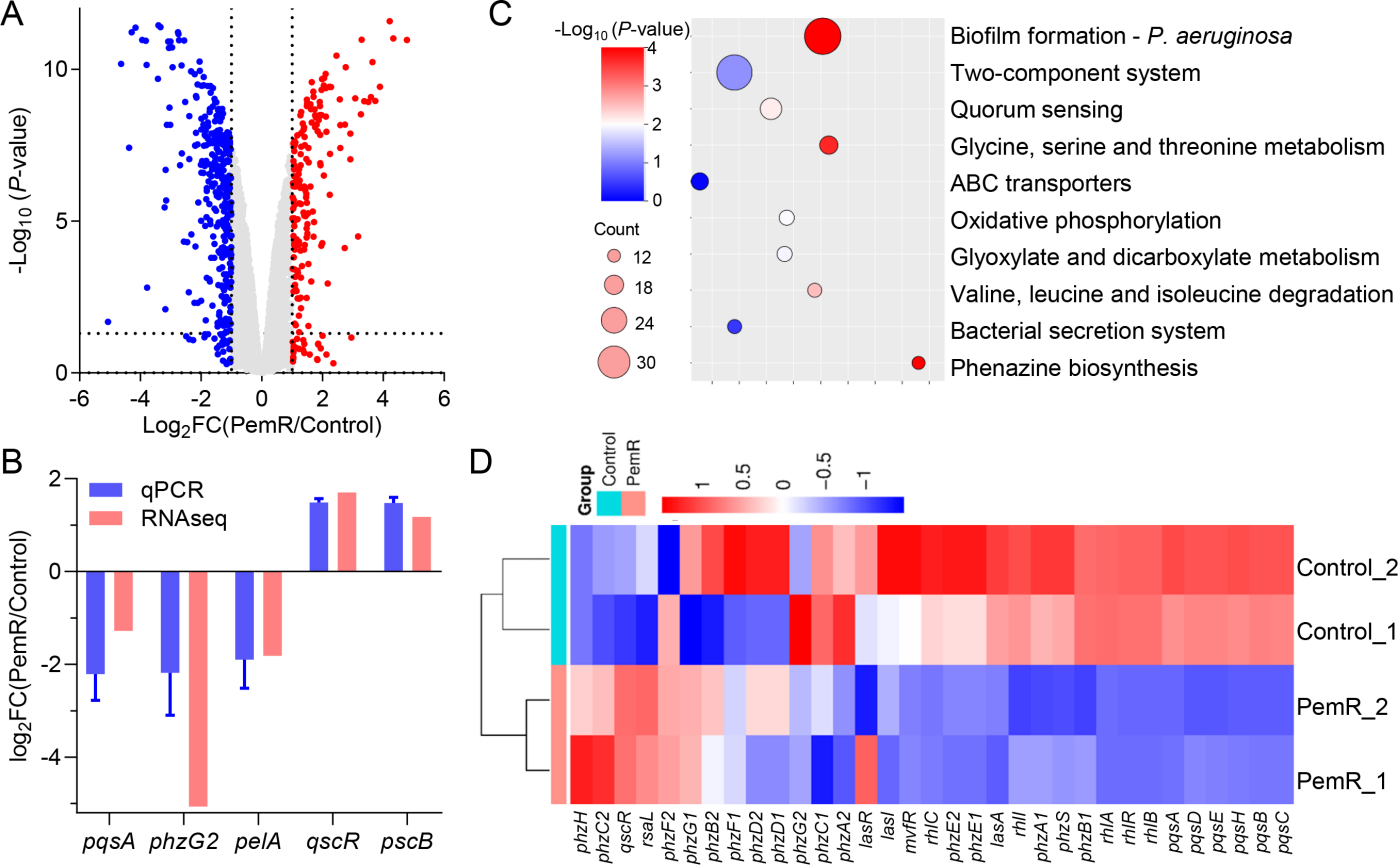
Global transcriptome analysis of expressing PemR in *P. aeruginosa*. (**A**) Transcriptomic analysis revealed differentially expressed genes (DEGs) between *P. aeruginosa* PAO1 carrying empty vector (Control) and *pemR*-expressing strain (PemR) through volcano plot analysis. Red and blue dots represent significantly up- and downregulated genes, respectively. The horizontal dashed line indicates the *P*-value cutoff (*P* = 0.05), while vertical dashed lines denote Log_2_FC thresholds (−1 and 1). (**B**) Validation of RNA-Seq data by qRT-PCR. Five randomly selected genes were analyzed using qRT-PCR to confirm the reliability of transcriptome results. Data are presented as mean ± SD from three independent experiments. (**C**) GO functional enrichment of PemR-associated DEGs. The top 10 significantly enriched terms (Fisher’s exact test, *P* < 0.001, FDR corrected) are shown. Circle area scales with DEG number; color gradient indicates statistical significance (−log_10_ adjusted *P*-value). (**D**) Heatmap of gene expression levels (RPKM) in PemR-expressing strains (PemR_1-2) versus empty vector controls (Control_1-2). Color gradient represents RPKM values across three biological replicates per strain (blue: low expression; red: high expression). Displayed genes are associated with the QS systems.

To further investigate the global impact of PemR on *P. aeruginosa*, transcriptomic profiling reveals genome-wide regulatory effects of PemR in *P. aeruginosa*. RNA-seq analysis of isogenic PemR-expressing PAO1 (PemR) versus wild-type PAO1 (Control), processed through DESeq2 with stringent thresholds (*P* < 0.05, |log_2_FC| > 1), identified 485 significantly altered genes: 166 upregulated and 319 downregulated (Table S3). These data demonstrate PemR’s extensive influence on the *P. aeruginosa* transcriptome, affecting 8.7% of coding genes (Fig. 4A). Validation of RNA-seq data by qRT-PCR confirmed the reliability of our transcriptomic analysis. Randomly selected genes showed consistent expression patterns between qRT-PCR and RNA-seq results (Fig. 4B). GO enrichment analysis of differentially expressed genes (DEGs) (*P* < 0.05) identified the top 10 impacted biological processes, visualized via bubble plot (circle size: gene count; color: -log_10_[*P*-value]) (Fig. 4C). These significantly enriched pathways—including biofilm formation, QS system, bacterial secretion systems, and phenazine biosynthesis—align precisely with the observed phenotypic changes. As *mvfR* is a central component of the QS system, we further validated PemR-mediated suppression of *mvfR* through heatmap analysis of QS-related gene expression patterns (Fig. 4D). A comparison of the PemR-regulated transcriptome with published data from a Δ*mvfR* mutant identified a significant overlap, notably among downregulated PQS pathway genes (Table S4) (40–42). This shared profile strongly supports the conclusion that PemR exerts its effects primarily through the inhibition of MvfR. Taken together, transcriptomic profiling confirms that PemR exerts widespread regulatory effects, particularly by downregulating genes involved in QS system, biofilm formation, and virulence, consistent with the observed phenotypic attenuation.

### PemR attenuates type VI secretion system in *P. aeruginosa* by promoting *rsmA* expression

**Fig 5:**
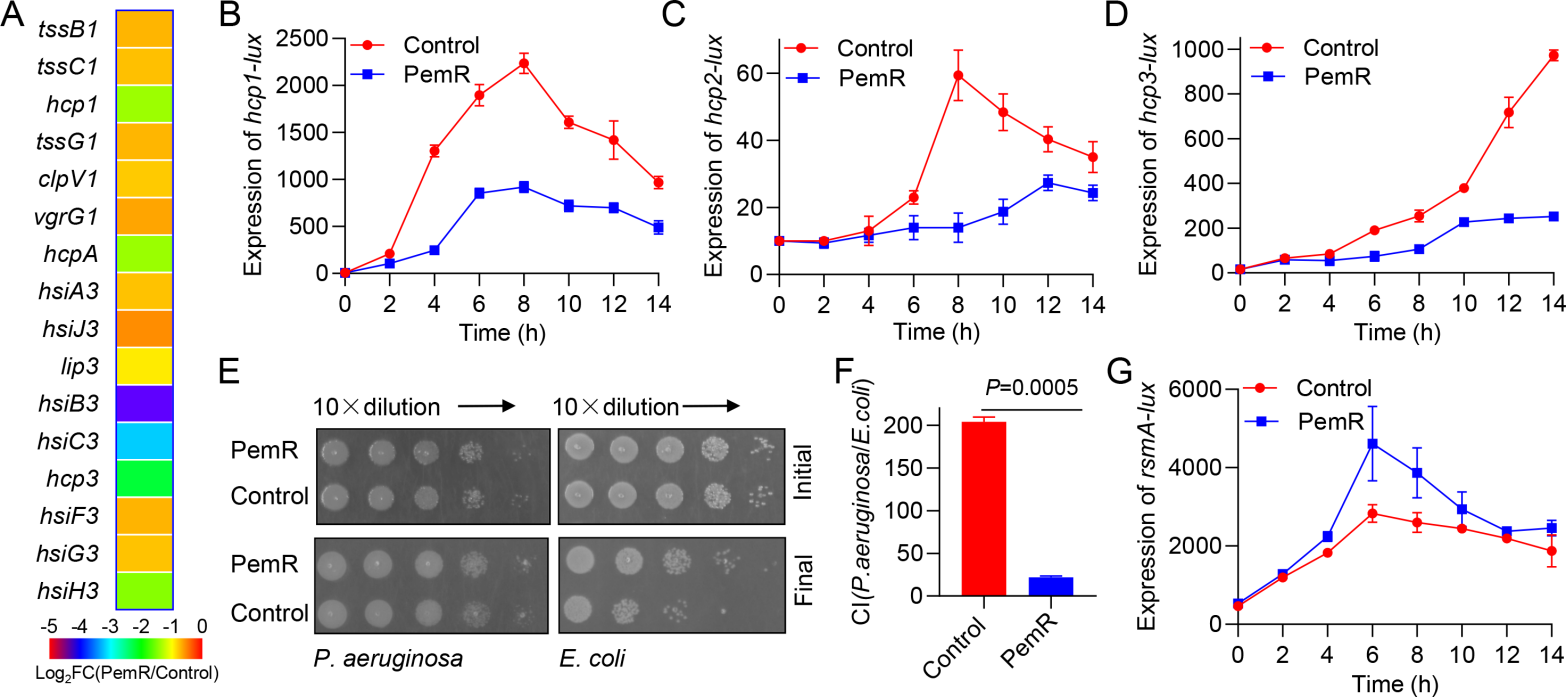
PemR represses type VI secretion system by promoting *rsmA* expression. (**A**) Comparative transcriptomic analysis revealed significant downregulation of T6SS-associated genes in *pemR*-expressing PAO1 (PemR) compared to the wild-type control (Control). (**B–D**) PemR suppresses *hcp1*, *hcp2,* and *hcp3* expression. Promoter activities of *hcp1*, *hcp2,* and *hcp3* were measured in PAO1 (Control) versus PAO1 expressing pemR (PemR). (**E**) Co-culture survival analysis of *E. coli* with PAO1 (Control) or PAO1 expressing pemR (PemR). Bacterial viability was assessed after 12-hour co-culture at an initial 5:1 ratio (*P. aeruginosa*/*E. coli*). (**F**) Competition Index (CI) = (Post-competition CFU ratio of *P. aeruginosa* to *E. coli*) / (Pre-competition CFU ratio of *P. aeruginosa* to *E. coli*). (**G**) PemR enhances *rsmA* expression. Promoter activity of *rsmA* was measured in PAO1 (Control) versus PAO1 expressing PemR (PemR). (**B–D and G**) Data shown are representative from three independent experiments. Error bars indicate mean ± standard deviation. Statistical significance was calculated using paired *T* test. (**E and F**) Data are representative of two independent replicates. Error bars indicate mean ± standard deviation. Statistical significance was calculated using paired *t*-test.

Transcriptomic analysis revealed significant downregulation of multiple T6SS-associated genes in PemR-expressing PAO1 (PemR) compared to wild-type PAO1 (Control), including *tssB1, hcp1*, *hcpA*, *hcp3*, and *hsiH3* (Fig. 5A) (43). Transcriptional profiling reveals T6SS suppression by PemR in *P. aeruginosa*. Promoter activity assays of hallmark T6SS effector genes (*hcp1, hcp2*, *hcp3*) demonstrated significant inhibition across all three secretion systems in PemR-expressing PAO1 (PemR), indicating broad-spectrum repression of bacterial weaponry (Fig. 5B through D). The T6SS serves as a central virulence determinant in pathogenic Gram-negative bacteria, translocating cytotoxic effectors into both microbial competitors and host cells during infection. To further validate the hypothesis that PemR attenuates T6SS-mediated antibacterial activity in *P. aeruginosa*, we co-cultured PemR-expressing PAO1 (PemR) and wild-type PAO1 (Control) with *E. coli* TG1 to assess their antimicrobial capacity. As shown in Fig. 5E, viable *E. coli* counts were significantly reduced after co-culture with the wild-type PAO1 (Control), while the competition index of the PemR-expressing PAO1 (PemR) decreased 10-fold (Fig. 5E and F).

In *P. aeruginosa*, the RsmA protein serves as a pivotal post-transcriptional repressor that directly binds to and inhibits the translation of T6SS core component mRNAs (e.g., *hcp*, *vgrG)*, thereby negatively regulating T6SS expression (44). We hypothesized that PemR regulates *rsmA* expression to suppress T6SS, so we constructed an *rsmA-lux* reporter and measured its promoter activity in PemR-expressing strains. The results showed that PemR enhanced *rsmA* expression, indicating that PemR positively regulates *rsmA* levels, thereby affecting T6SS-mediated antibacterial activity in *P. aeruginosa* (Fig. 5G). Thus, beyond dampening QS-controlled virulence, PemR also impairs bacterial competitiveness by upregulating *rsmA* to suppress T6SS expression and function, further compromising *P. aeruginosa* fitness.

### PemR reduces motility in *P. aeruginosa*

**Fig 6:**
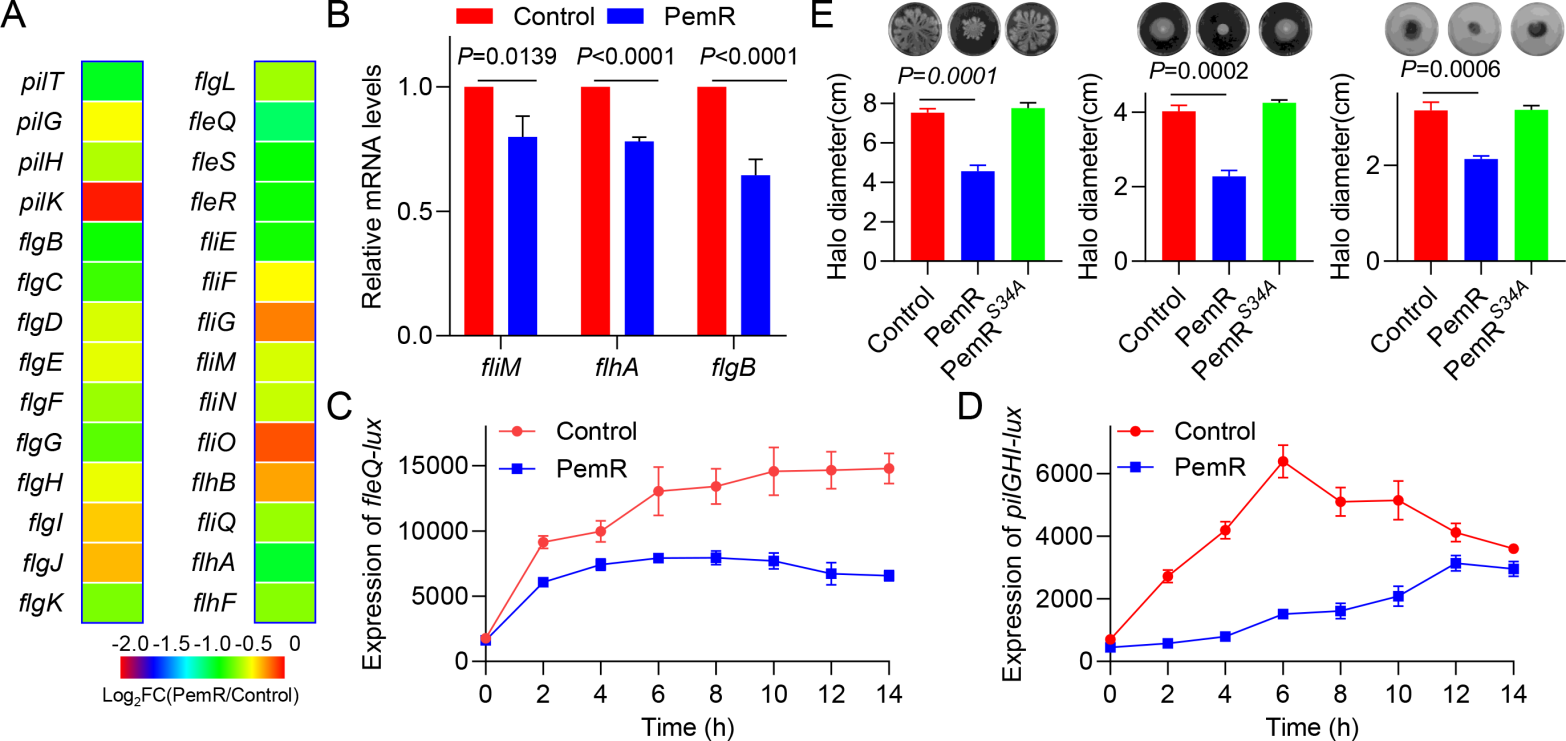
PemR reduces motility in *P. aeruginosa*. (**A**) Comparative transcriptomics revealed significantly reduced expression of flagella- and pilus-associated genes in *pemR*-expressing PAO1 (PemR) versus wild-type PAO1 (Control). (**B**) qRT-PCR analysis of *fliM*, *flhA*, and *flaB* mRNA levels in PAO1 (Control) versus PemR-expressing PAO1 (PemR). (**C–D**) PemR suppresses *pilG* and *fleQ* expression. Promoter activities of *pilG* and *fleQ* were measured in PAO1 (Control) versus PAO1 expressing PemR (PemR). (**E**) Motility assays: PAO1 (Control), PAO1 (PemR), and PAO1 (PemR^S34A^) strains grown to OD_600_=2.0 were spotted onto swimming, swarming, and twitching agar plates. Incubation was performed at 30°C for 16 h (swimming) or 37°C for 16 h (swarming and twitching). (**B–D**) Data shown are representative from three independent experiments. Error bars indicate mean ± standard deviation. Statistical significance was calculated using paired *t*-test. (**E**) Data are representative of three independent replicates. Error bars indicate mean ± standard deviation. Statistical significance was calculated using paired *t*-test.

Motility is a key virulence trait of *P. aeruginosa*, essential for environmental adaptation, host colonization, and biofilm development. The bacterium employs three distinct motility mechanisms: flagellum-driven swimming, cooperative swarming, and type IV pilus-mediated twitching (45, 46). Transcriptomic profiling demonstrated a significant downregulation of flagellar and type IV pilus-associated genes (*flgB*, *flgC*, *flgD*, *flgE*, *flgF*, *flgG*, *flgH*, *flgI*, *flgJ*, *flhA*, *pilG*, *pilT*, and *pilH*) in PemR-expressing strains, suggesting a regulatory role of PemR in bacterial motility apparatus assembly (Fig. 6A). To validate these results, three genes were selected for RT-qPCR analysis. The data showed that the expression levels of *fliM*, *flhA*, and *flgB* were significantly lower in the PemR-expressing strain compared to the control strain (Fig. 6B). Simultaneously, we constructed transcriptional reporter strains (*pilGHI-lux* and *fleQ-lux*) for real-time monitoring of promoter activity. Time-course experiments revealed significantly lower luminescence signals in PemR-overexpressing strains compared to wild-type controls throughout bacterial growth, demonstrating that PemR negatively regulates motility in *P. aeruginosa* (Fig. 6C and D). To further elucidate the functional role of PemR in *P. aeruginosa* motility, we conducted comprehensive motility assays, including swimming, swarming, and twitching analyses. Strikingly, PemR-expressing strains exhibited significantly reduced motility zones compared to wild-type controls (*P* < 0.001), demonstrating that PemR acts as a potent negative regulator of *P. aeruginosa* motility. However, PAO1 expressing PemR^S34A^ exhibited a comparable motility ability to the control strain (Fig. 6E). In summary, PemR comprehensively inhibits *P. aeruginosa* motility—swimming, swarming, and twitching—by downregulating flagellar and pilus gene expression, thereby limiting another critical virulence-associated behavior.

## DISCUSSION

**Fig 7:**
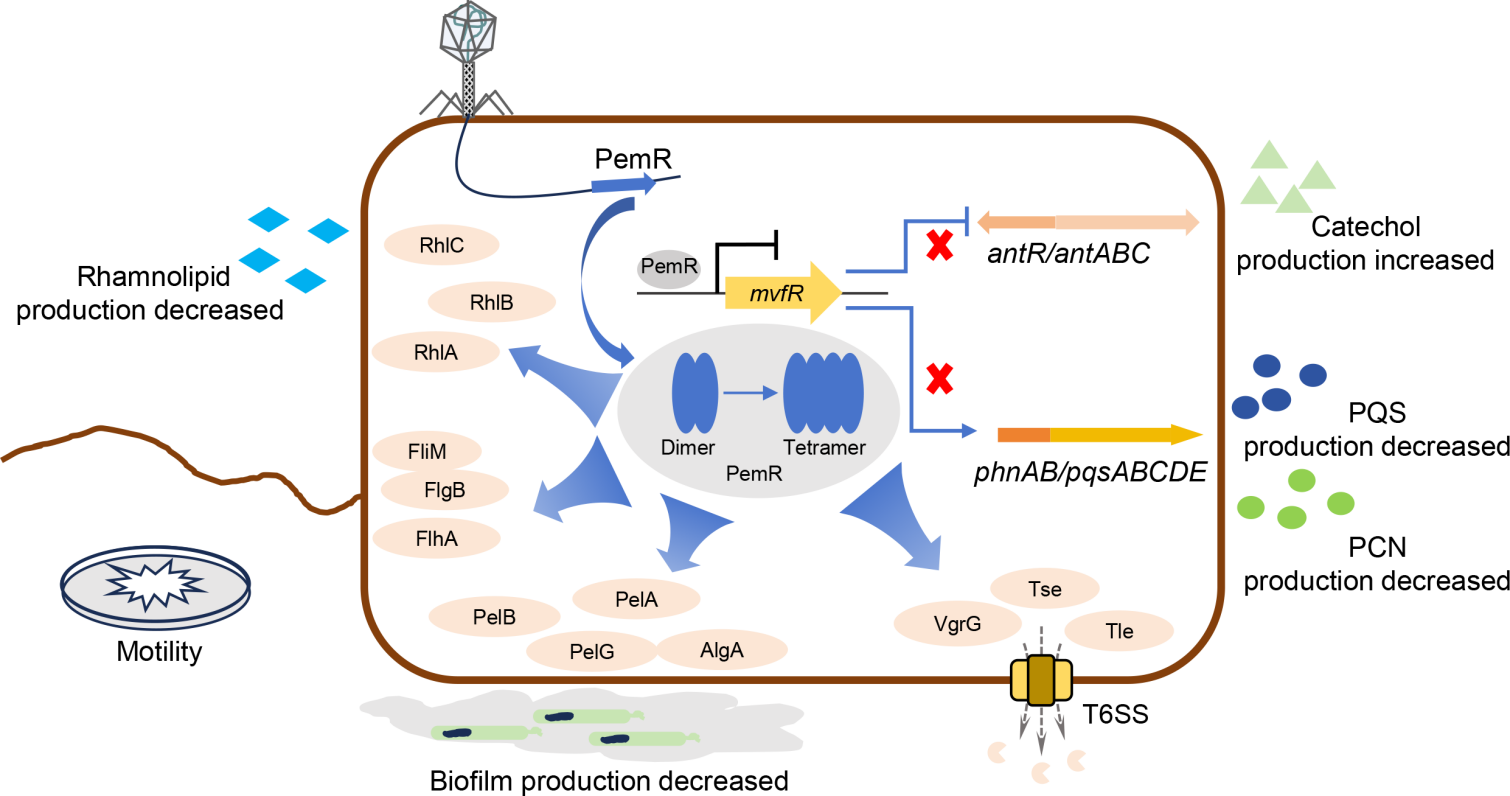
Proposed model of PemR-regulated pathway in *P. aeruginosa*. The phage hijacks the host translational machinery to express the transcriptional regulator PemR, after infection of *P. aeruginosa* by bacteriophage PAYQ66. PemR is a pivotal factor that modulates multiple cellular pathways in *P. aeruginosa*, including PQS and catechol synthesis, pyocyanin production, rhamnolipid synthesis, biofilm formation, swarming motility, and T6SS-mediated antibacterial activity. Black arrows denote direct regulatory interactions between PemR and its target genes; blue arrows indicate indirect regulatory relationships. PemR represses *mvfR* transcription by directly binding to its promoter region, thereby controlling PQS and catechol biosynthesis. Other cellular processes are modulated indirectly via PemR-dependent changes in the expression levels of genes involved in diverse physiological pathways. FlgB, FlhA, FliM, and PilG are key proteins for flagellum and pilus assembly and motility. RhlA, RhlB, and RhlC are essential enzymes for rhamnolipid synthesis. VgrG, Tle, and Tse are core components required for T6SS-mediated antibacterial activity. PelA, PelB, and PelC are critical enzymes for biofilm formation.

*P. aeruginosa*, a formidable opportunistic pathogen, poses escalating clinical challenges due to its extraordinary capacity for developing multidrug resistance and robust biofilm formation. This study reports the functional characterization of PemR, a novel transcriptional regulator derived from lytic phage PAYQ66 that orchestrates multimodal attenuation of *P. aeruginosa* virulence (Fig. 1). Our findings establish PemR as a master epigenetic modulator that fundamentally rewires host metabolic and virulence networks through three synergistic mechanisms: (i) direct transcriptional repression of the QS master regulator *mvfR*; (ii) global perturbation of QS-controlled virulence pathways; and (iii) suppression of T6SS-mediated interbacterial competition via RsmA upregulation (Fig. 7).

PemR, a phage-encoded transcriptional regulator, attenuates *P. aeruginosa* virulence by directly repressing *mvfR* and rewiring host metabolism and virulence networks. Biochemical analyses revealed that PemR forms both dimeric and tetrameric oligomers, as demonstrated by SEC (Fig. 1F), and both oligomeric states are capable of binding the *mvfR* promoter region with high specificity, as evidenced by EMSA assays (Fig. 1G; Fig. S8A and B). This oligomerization, mediated by a conserved HTH domain, is essential for DNA recognition and transcriptional repression, as the PemR^S34A^ mutant lacking this domain fails to oligomerize or bind DNA (Fig. 1H and I). While PemR represents a distinct mechanism of phage interference with bacterial QS, unlike previously reported effectors such as Aqs1 and PIT2 that target LasR, it is noteworthy that phage-encoded transcriptional regulators have also been shown to enhance bacterial virulence in other systems (31, 32). For example, the Cro regulator from a Stx-lambdoid-like phage in enterohemorrhagic *E. coli* directly activates host genes encoding the type III secretion system, thereby enhancing virulence even during lysogeny (47). Thus, PemR adds to a growing repertoire of phage-encoded transcriptional hijackers that can either potentiate or attenuate bacterial virulence, highlighting the multifaceted role of phages in shaping host pathogenicity. Rather than representing a fundamental paradigm shift, PemR exemplifies the evolutionary versatility of phage-host interactions and underscores the potential of phage-derived regulators as tools for anti-virulence strategies. Notably, the interplay between phage-encoded proteins and host QS is intricate. Studies have found that the QS system LasR/I upregulates *galU* expression, enhancing lipopolysaccharide (LPS) synthesis and thereby increasing phage adsorption and infection efficiency in *P. aeruginosa* (48). This contrasts with PemR, which exploits QS repression to attenuate virulence while reprogramming host metabolism to serve the phage’s own interests.

Crucially, PemR’s dual-targeting strategy simultaneously suppresses *mvfR* and enhances *rsmA* expression, paralyzing both acute virulence (pyocyanin, rhamnolipids, biofilm formation, and motility) and ecological competitiveness (T6SS-mediated antagonism) (Fig. 3 and 5). This multi-layered intervention likely provides multiple selective advantages for phage propagation. Beyond resource reallocation, phage-encoded protein PemR suppresses the T6SS, an energetically expensive weapon whose assembly consumes copious ATP, thereby diverting host resources and energy toward phage replication and particle construction (43). Meanwhile, PemR inhibits bacterial virulence, which could delay immune-mediated clearance of its host, thereby affording itself a longer replication window (49, 50). Finally, in polymicrobial communities, disarming the host T6SS erodes that bacterium’s competitive edge, which is often mediated by tit-for-tat interactions (51, 52). This reduction in competitiveness may facilitate phage dissemination and long-term persistence within the mixed population of phage host.

Transcriptomic profiling confirmed PemR’s global regulatory influence, with differentially expressed genes enriched in virulence-associated pathways (Fig. 4). The coordinate downregulation of *phzA1/A2* (pyocyanin), *rhlAB* (rhamnolipids), and *pelA* (biofilm matrix) aligns with observed phenotypic attenuation (Fig. 3). Notably, the 90% reduction in pyocyanin may directly contribute to enhanced host survival in *Galleria mellonella* infection models (78% vs 0% survival) (Fig. 3H and I). Equally significant is the T6SS suppression mediated through RsmA upregulation (Fig. 5). In addition to direct transcriptional repression, PemR also indirectly modulates bacterial motility by downregulating flagellar and type IV pilus genes without directly binding to their promoters (Fig. 6; Fig. S8C and D), further underscoring its role as a global modulator that attenuates virulence through both direct and indirect pathways. Additionally, phylogenetic analysis reveals that PemR homologs are present across diverse bacteriophages (Fig. S9). These homologs, identified from public databases, cluster within multiple phage lineages, including members of *Caudovirales*, suggesting that PemR represents a conserved phage-encoded transcriptional regulator. Some homologs originate from lytic phages isolated from clinical or environmental samples, implying a broad role in modulating host virulence. Further study of these homologs may reveal common mechanisms in phage-mediated virulence regulation and support anti-virulence therapeutic development.

PemR exemplifies the untapped potential of phage-encoded anti-virulence effectors. Its ability to attenuate pathogenicity without bactericidal pressure aligns with next-generation anti-virulence paradigms that minimize resistance selection (53). EMSA of the PemR-*mvfR* promoter complex (Fig. 1D through G) provides a blueprint for rational design of *mvfR*-targeting anti-virulence compounds, enabling precise disruption of this key QS control node. The observed *in vivo* efficacy in plant and insect models warrants investigation in mammalian systems and provides a theoretical basis for the development of novel antimicrobial peptides (54).

This study reveals that the phage-encoded virulence inhibitor PemR attenuates the pathogenicity of *P. aeruginosa*. Although we have confirmed that PemR can reduce bacterial virulence by hijacking the MvfR regulatory network, potential counter-defense strategies that bacteria may evolve remain to be explored. It should be noted that the constitutive overexpression system used in this study, while advantageous for mechanistic analysis, may not fully capture the transient expression characteristics of *pemR* during natural phage infection. Therefore, future work should focus on quantitatively validating the dynamic expression and function of PemR during infection, as well as investigating possible host adaptation mechanisms, which are essential for assessing the durability of PemR-inspired anti-virulence strategies.

## MATERIALS AND METHODS

### Bacterial strains and growth conditions

The bacterial strains and plasmids utilized in this study are detailed in Table S2. PAO1 and its derivatives were cultured at 37°C in Luria-Bertani (LB) medium (1% tryptone, 0.5% yeast extract, 1% NaCl) or on *Pseudomonas* isolation agar (PIA), supplemented with appropriate antibiotics to ensure plasmid retention. *E. coli* strains were also cultured at 37°C in LB medium, with antibiotic concentrations set at 50 μg/mL kanamycin, 10 μg/mL tetracycline, and 100 μg/mL carbenicillin for *E. coli*, and 100 μg/mL tetracycline, 1200 μg/mL trimethoprim, and 300 μg/mL carbenicillin for *P. aeruginosa.*

### Construction of plasmids and measurement of promoter activity

The primers employed in this study are listed in Table S5. To construct expression plasmids, the open reading frame (ORF) fragment of phage PAYQ66_*orf47* (*pemR*) was amplified by PCR using specific primers. The amplified DNA fragment and the target vector plasmids (pME6032 or pET28a) were digested with the appropriate restriction enzymes and ligated, resulting in the corresponding plasmid derivatives.

Plasmid pMS402 and mini-CTX-*luxCDABE*, which carries a promoterless *luxCDABE* operon, was used to generate promoter-*luxCDABE* reporter constructs. The *mvfR* promoter region was amplified via PCR and cloned into mini-CTX-*luxCDABE* to create the *mvfR*-lux reporter plasmid. Other promoter–*luxCDABE* constructs were generated using the same cloning strategy, and all plasmids were verified through DNA sequencing.

To screen for phage genes that regulate *mvfR* expression, we constructed a transcriptional reporter strain in which the promoter region of *mvfR* was fused to a promoter-less *luxCDABE* bioluminescence operon (*mini-mvfR-lux*) and integrated into the genome of *P. aeruginosa* PAO1. In this system, the intensity of the luminescence signal directly reflects the transcriptional activity of the *mvfR* promoter. Luminescence was recorded both qualitatively, as colony images captured with a chemiluminescence imager, and quantitatively, as counts per second (CPS) measured using a microplate luminometer (BioTek Synergy 2).

### Measurement of promoter activities by *lux* fusions

Expression of the lux-based reporters was quantified as counts per second of emitted light. Overnight cultures of the reporter strains were diluted to an OD_600_ of 0.2 in fresh LB medium and incubated for an additional 2 h at 37°C. The cultures were then diluted 1:20 in fresh LB medium and transferred into black, transparent-bottom 96-well plates. Fifty microliters of sterile mineral oil were added to each well to prevent evaporation. Promoter activity and bacterial growth (OD_600_) were continuously measured at 30-minute intervals over 24 h using a BioTek Synergy 2 plate reader.

### Western blot assay

Overnight bacterial cultures were inoculated at 1% into fresh LB medium and sub-cultured with shaking at 37°C until mid-exponential phase (OD_600_ = 1.5). Cell pellets from 0.5 mL cultures were harvested by centrifugation (12,000 × *g*, 2 min, 4°C) and resuspended in 50 μL of 1× SDS loading buffer. Following denaturation at 100°C for 10 min, 5 μL aliquots of soluble protein extracts were resolved through 15% SDS-polyacrylamide gel electrophoresis and transferred onto polyvinylidene difluoride (PVDF) membranes (Millipore, Billerica, MA). After blocking with 5% skimmed milk in TBST (20 mM Tris-HCl, 150 mM NaCl, 0.1% Tween 20, pH 7.6) for 1 h at room temperature, membranes were incubated overnight at 4°C with primary antibodies: either mouse monoclonal anti-Flag (1:5,000) or rabbit polyclonal anti-RNA polymerase α subunit (1:3,000). Following three washes with TBST, membranes were probed with horseradish peroxidase (HRP)-conjugated goat anti-mouse/anti-rabbit IgG secondary antibodies (1:10,000) for 1 h at room temperature. Protein signals were detected using an ECL Plus Chemiluminescence Substrate (Tanon Science & Technology, Shanghai, China) and imaged with a Tanon 5200 Multi-Imaging System under standardized auto-exposure protocols.

### PQS production assay

PQS extraction and analysis were performed as follows: overnight bacterial cultures were diluted 100-fold into fresh LB medium and incubated at 37°C for 24 h. After incubation, 500 μL of the culture was mixed with 1 mL of acidified ethyl acetate, vortexed for 2 min, and centrifuged at 16,000 × *g* for 10 min. The upper organic phase was transferred to a new tube and air-dried at room temperature overnight. The dried extracts were reconstituted in 50 μL of a solvent containing a 1:1 mixture of acidified ethyl acetate and acetonitrile. Samples were analyzed by thin-layer chromatography (TLC), and their densities were quantified using ImageJ software.

### Electrophoretic mobility shift assay (EMSA)

Binding reactions containing PemR protein and 2.0 ng/μL *mvfR* promoter (−1 to +746 relative to the transcription start site of *mvfR*) probe in 20 μL gel shift buffer (10 mM Tris, 10 mM MgCl₂, 1 mM DTT, 5 μg/mL sheared salmon sperm DNA, pH 7.5) were prepared. The *rhlR* promoter probe served as control. After a 30-minute incubation at 25°C, reaction aliquots were resolved through non-denaturing 6% polyacrylamide gels in 0.5× TBE buffer (45 mM Tris-borate, 1 mM EDTA) at 100 V for 120 min at 4°C, and the DNA probe was detected using SYBR Green.

### Quantitative real-time PCR

*P. aeruginosa* cultures were harvested at medium phase (OD_600_ = 1.0). Total RNA was extracted using the RNAprep Pure Cell/Bacteria Kit (TIANGEN, Beijing, China), followed by on-column DNase I treatment (RNase-free), according to the manufacturer’s instructions. The integrity of the isolated RNA was assessed using 1.2% denaturing agarose gel electrophoresis, and purity and concentration were determined spectrophotometrically with a NanoDrop 2000 spectrophotometer (Thermo Scientific). First-strand cDNA synthesis was performed with 1 μg of total RNA using the TransScript First-Strand cDNA Synthesis SuperMix (TransGen Biotech, Beijing, China). qRT-PCR was conducted in triplicate 20 μL reactions using TransStart Green qPCR SuperMix (TransGen Biotech) on a CFX96 Real-Time PCR Detection System (Bio-Rad). All primer pairs (Table S2) were validated with cycling parameters: initial denaturation at 95°C for 30 s, followed by 40 cycles of denaturation at 94°C for 15 s, annealing at primer-specific temperature (Tm) for 30 s, and extension at 72°C for 30 s. The 16S rRNA gene served as an endogenous control for normalization. The relative changes in gene expression were calculated using the comparative quantification method.

### Biofilm assay

An overnight culture was diluted 1:100 into borosilicate glass tubes containing 3 mL of fresh LB broth. The tubes were incubated statically at 25°C for 19 h to allow biofilm formation. Following incubation, planktonic bacteria were gently removed, and the tubes were washed three times with sterile water to eliminate non-adherent cells. The remaining biofilm was stained with 0.1% crystal violet at room temperature for 20 min. Excess dye was removed, and the tubes were air-dried. The stained biofilm was solubilized with 95% absolute ethanol, and absorbance at OD_600_ was measured using a microplate reader Synergy 2X (BioTek, USA).

### RNA-seq and data analysis

Bacterial strains were cultured in LB medium at 37°C until the OD_600_ reached approximately 1.0. Total RNA was extracted immediately after cell harvesting using the TruSeq Stranded Total RNA Library Prep Kit, according to the manufacturer’s instructions. cDNA libraries were constructed through reverse transcription, followed by end repair, adapter ligation, and PCR amplification. The resulting cDNA libraries underwent high-throughput sequencing on the Illumina NovaSeq 6000 platform (Majorbio-Shanghai). Three biological replicates were performed for each RNA sequencing sample to ensure experimental reproducibility. Sequencing reads were aligned to the reference genome of PAO1 (NC_002516.2) using Bowtie 2, retaining only uniquely mapped reads for downstream analysis. DEGs were identified using DESeq2, with adjusted *P*-values (Benjamini-Hochberg method) < 0.05 and |log₂ fold change| > 1, comparing PAO1 (PemR) with the PAO1 strain. Identified DEGs were subjected to Kyoto Encyclopedia of Genes and Genomes (KEGG) pathway enrichment analysis.

### Motility assay

For swimming motility, media containing 0.3% agar, 1% tryptone, and 0.5% NaCl were prepared. *P. aeruginosa* cultures were adjusted to OD_600_ = 1.0, and 2 μL were centrally spotted onto the agar, then incubated at 30°C for 16 h. Swarming assays utilized media with 0.5% agar, 0.8% nutrient broth, and 0.5% glucose, with cultures similarly prepared and incubated at 37°C for 16 h. For twitching motility, *P. aeruginosa* cultures were diluted to OD_600_ = 1.0. Two microliters were spotted onto LB agar with 1% agar, and the plates were incubated at 37°C for 24 h after inoculum absorption. Images were captured using the Tanon 2500 imaging system.

### Bacterial two-hybrid assay

To investigate whether PemR functions as a dimer, a bacterial two-hybrid (BACTH) system was employed. The PemR coding sequence was cloned into either the pKT25 or pUT18C plasmid and co-transformed into competent *E. coli* BTH101. The co-transformed cells were plated on LB agar supplemented with kanamycin (50 μg/mL), ampicillin (100 μg/mL), X-gal (20 μg/mL), and IPTG (0.5 mM) and incubated at 30°C for 24 h. Cells containing pKT25-*zip*/pUT18C-*zip* served as a positive control, while those carrying pKT25/pUT18C-PemR and pUT18C/pKT25-PemR were used as negative controls.

### *Galleria mellonella* killing assay

The *Galleria mellonella* infection model was employed as an invertebrate model system. *P. aeruginosa* was cultured in LB broth to an optical density of OD_600_ = 1.0, washed three times with sterile phosphate-buffered saline (PBS), and resuspended in PBS at a final concentration of 10⁴ CFU/mL. Using a 100 μL Hamilton syringe, 20 μL aliquots of either PBS, PAO1 (Control), and PAO1 expressing PemR (PemR) bacterial suspensions were injected into the last left proleg of each *G. mellonella* larva. Infected larvae were maintained in a dark incubator at 37°C with continuous monitoring. Mortality was recorded at designated time points, with death defined as the absence of response to gentle tactile stimulation.

### Chinese cabbage infection assay

Chinese cabbage infection was determined as described previously (38, 39). *P. aeruginosa* was cultured overnight at 37°C until it reached the logarithmic growth phase. The bacteria were harvested by centrifugation at 4,000 × *g* for 5 min, and the supernatant was discarded. The bacterial pellet was washed with 10 mM PBS and resuspended in 10 mM PBS, adjusting the OD_600_ to 1.0. Cabbage stems were treated with 0.1% H_2_O_2_ solution for disinfection and placed on filter paper soaked in 10 mM MgSO₄. Subsequently, 10 μL of the bacterial suspension was injected into each cabbage stem using a sterile micropipette, and the samples were incubated at 30°C for 6 days. Growth status and rot degree of the cabbage were regularly observed, and results were recorded using a scoring system from no rot to complete rot. Finally, data were organized to assess the pathogenicity of *P. aeruginosa* on cabbage.

### Expression and purification of protein

The pET28a-PemR plasmid was introduced into *E. coli* BL21 by transformation to establish a recombinant strain for PemR expression. Transformed *E. coli* BL21 were cultured in LB medium at 37°C with agitation until OD_600_ reached approximately 1.0. Recombinant protein expression was induced by adding IPTG to a final concentration of 1 mM, followed by incubation at 16°C for 16 h with continuous shaking. Post-induction, cells were harvested by centrifugation at 4,000 × *g* for 15 min at 4°C and ultrasonic crushing in His Buffer A (10 mM Tris-HCl, 500 mM NaCl, 10% glycerol, pH 7.5). Cell lysates were clarified by centrifugation at 12,000 × *g* for 30 min at 4°C, and the supernatant was applied to a Ni-NTA affinity chromatography column (GE Healthcare, Piscataway, NJ, USA). The column was extensively washed with His Buffer B (50 mM Tris-HCl, 500 mM NaCl, 500 mM imidazole, 10% glycerol, pH 7.5) to remove nonspecifically bound proteins. His6-tagged PemR was eluted using a linear gradient of His Buffer B, increasing imidazole concentration from 50 mM to 500 mM. Eluted fractions containing PemR protein were pooled and desalted into His Buffer C (50 mM Tris-HCl, 300 mM NaCl, 10% glycerol, pH 7.5) using a HiTrap Desalting column (GE Healthcare, Piscataway, NJ, USA). The purified protein was analyzed by sodium dodecyl sulfate-polyacrylamide gel electrophoresis (SDS-PAGE) to assess purity and integrity. Confirmed PemR protein samples were stored at −80°C until further use.

### Size exclusion chromatography

Size exclusion chromatography analysis was carried out using AKTA purification system with Seplife S-100 column (Sunresin Seplife, Suzhou, China). The column was first equilibrated with 20 mM Tris-HCl, 150 mM NaCl, pH 7.4, at 4°C, followed by the injection of 5 mg of purified PemR protein. Proteins were collected based on peak fractions, and the collected fractions were subjected to SDS-PAGE electrophoresis, followed by Coomassie Brilliant Blue analysis.

### Bacterial competition assay

In bacterial competition assays, overnight cultures of PemR-expressing PAO1 strains (PemR) and wild-type PAO1 strains (control), both carrying the pME6032 plasmid (conferring tetracycline resistance), were mixed at a 5:1 ratio with overnight cultures of *E. coli* carrying the pMS402 plasmid (conferring trimethoprim resistance). A 5 μL aliquot of the mixed bacterial suspension was spotted onto a nitrocellulose membrane overlaid on LB-LS agar. After 12 h of incubation at 37°C, the bacterial cells were resuspended in 5 mL of LB medium, serially diluted, and plated on LB agar plates containing tetracycline (Tc) or trimethoprim (Tmp) to determine the final colony-forming units (CFU). Colonies on Tc plates represented *P. aeruginosa*, while colonies on Tmp plates represented *E. coli*. Three independent biological replicates were performed for statistical analysis.

### Rhamnolipid production analysis

Rhamnolipid biosynthesis was evaluated using the CTAB-methylene blue agar plate assay. PemR-expressing PAO1 (PemR) and wild-type PAO1 (Control) were cultured overnight in LB broth and then sub-cultured into fresh medium to reach an OD_600_ of 1.0. Aliquots (2 μL) of each culture were spotted onto mineral salt agar plates supplemented with 0.2 mg/mL CTAB, 0.005 mg/mL methylene blue, and 20 g/L glycerol (pH 7.0). Plates were incubated at 30°C for 48 h, and following incubation, colony diameter and total halo diameter were measured using ImageJ software.

### Catechol production monitoring by HPLC

To monitor catechol production, cells were cultured overnight in LB medium at 37°C. One milliliter of culture was centrifuged at 12,000 × *g* for 3 min, and the supernatant was collected and filter-sterilized through a 0.22μm membrane. OD_600_ of the supernatants was adjusted to ensure consistent cell densities across samples. Analysis was performed using a C18 reverse-phase high-performance liquid chromatography (HPLC) column (Waters, Milford, MA, USA) with a flow rate of 1 mL/min. Mobile phase A consisted of 30% acetonitrile containing 0.1% phosphoric acid, and detection was carried out at 282 nm using a UV detector. Chromatographic peak areas were quantified with Shimadzu LabSolutions software (Shimadzu, Japan).

## Data Availability

The data that support the findings of this study are available in the supplemental material. The genome sequence of phage PAYQ66 has been deposited in GenBank under the accession number PQ072796.2. All sequencing data have been uploaded to BioProject under accession number PRJNA1302108.

## SUPPLEMENTAL MATERIAL

Supplemental figures jvi.02071-25-s0001.pdf Figures S1 to S9.

Supplemental tables jvi.02071-25-s0002.xlsx Tables S1 to S5.

ASM does not own the copyrights to Supplemental Material that may be linked to, or accessed through, an article. The authors have granted ASM a non-exclusive, world-wide license to publish the Supplemental Material files. Please contact the corresponding author directly for reuse.
