# Supplementary material for: The bacteriophage-encoded regulator PemR attenuates Pseudomonas aeruginosa virulence by hijacking quorum sensing and metabolism
Source: J Virol. 2026 Feb 5;100(3):e02071-25. doi: 10.1128/jvi.02071-25 (PMC13011342; doi:10.1128/jvi.02071-25)
Supplement: Supplemental figures — Figures S1 to S9. [file jvi.02071-25-s0001.pdf]

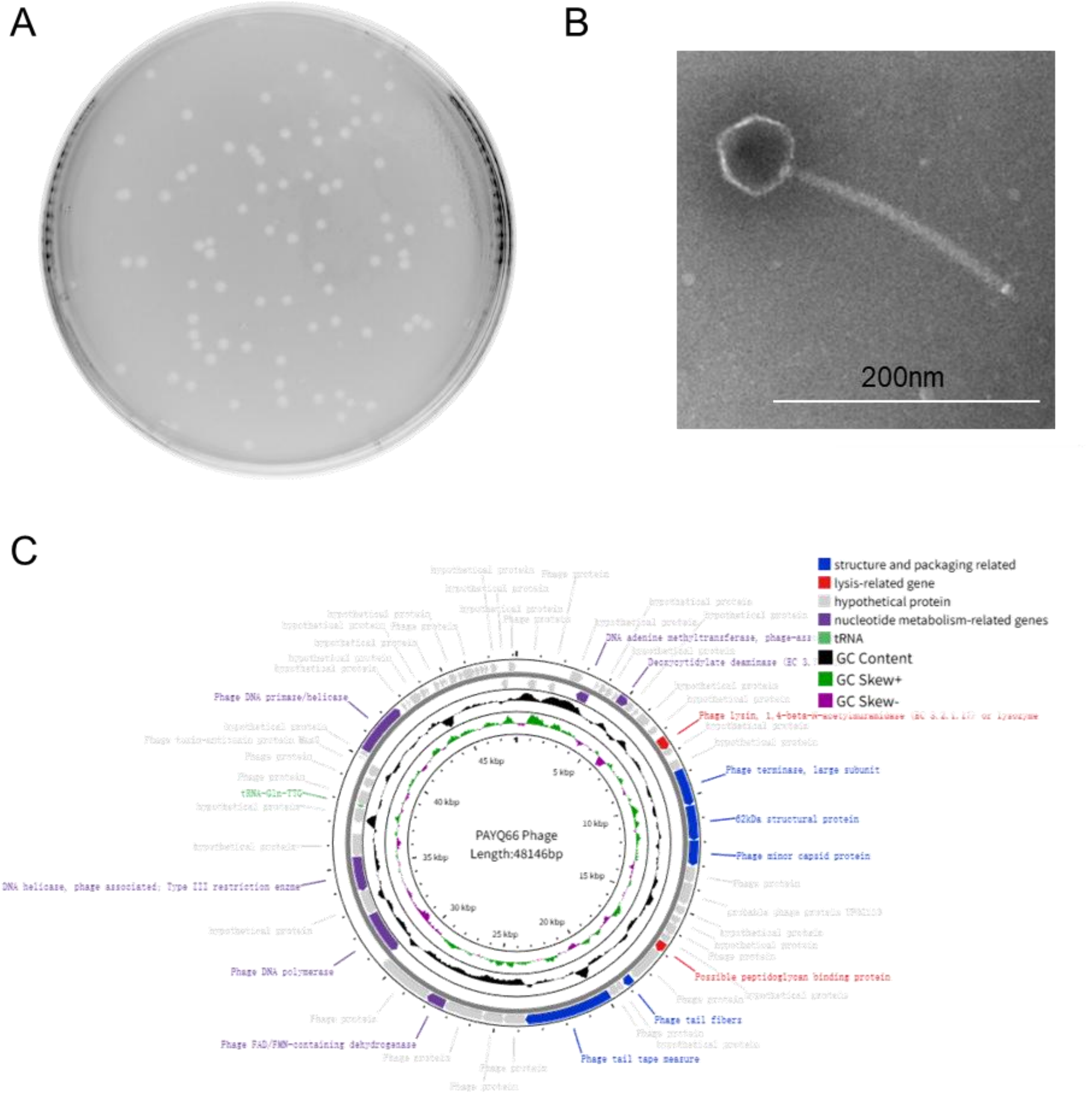

Fig. S1. (A) Small and clear plaques of PAYQ66 phage**. (B)** Electron micrograph of PAYQ66 phage particles. **(C)** Genome map of PAYQ66 phage. Specific colors indicate the ORFs according to their functional categories.

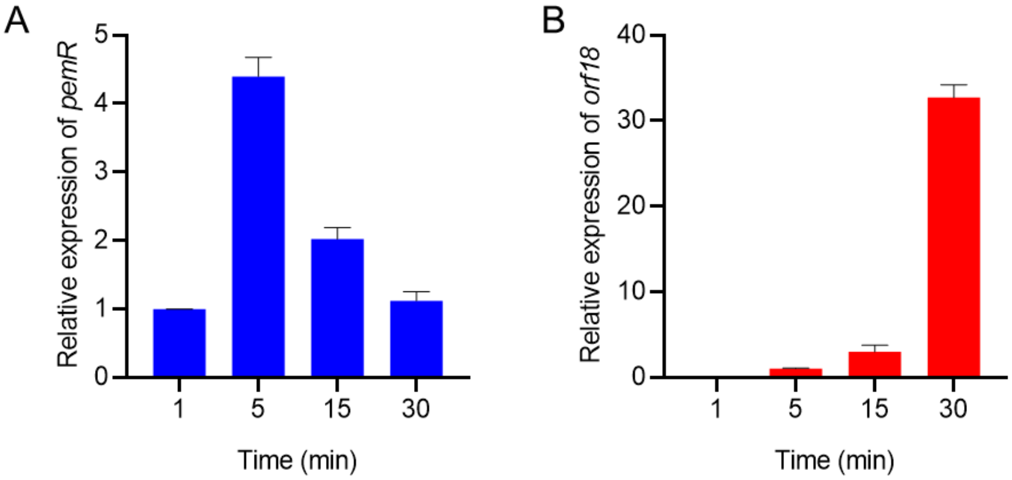

Fig. S2. The transcription of *pemR* **(A)** and *orf18* **(B)** was analyzed at 1, 5, 15, and 30 minutes post-infection (mpi). Data shown are representative from three independent experiments. Error bars indicate mean ± standard deviation.

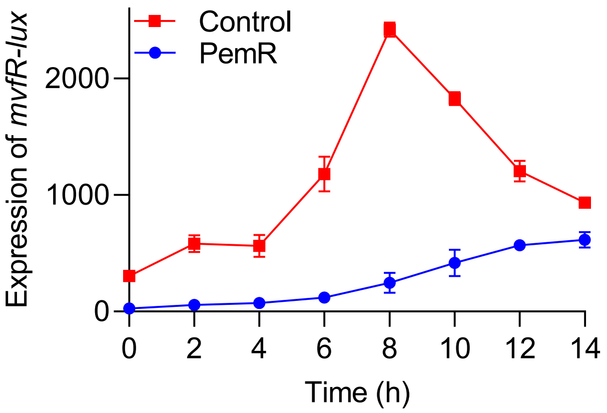

Fig. S3. PemR suppresses mvfR expression. Promoter activity of *mvfR* was measured in PAO1 (Control) versus PAO1 expressing *pemR* (PemR). Data are representative of three independent replicates. Error bars indicate mean ± standard deviation.

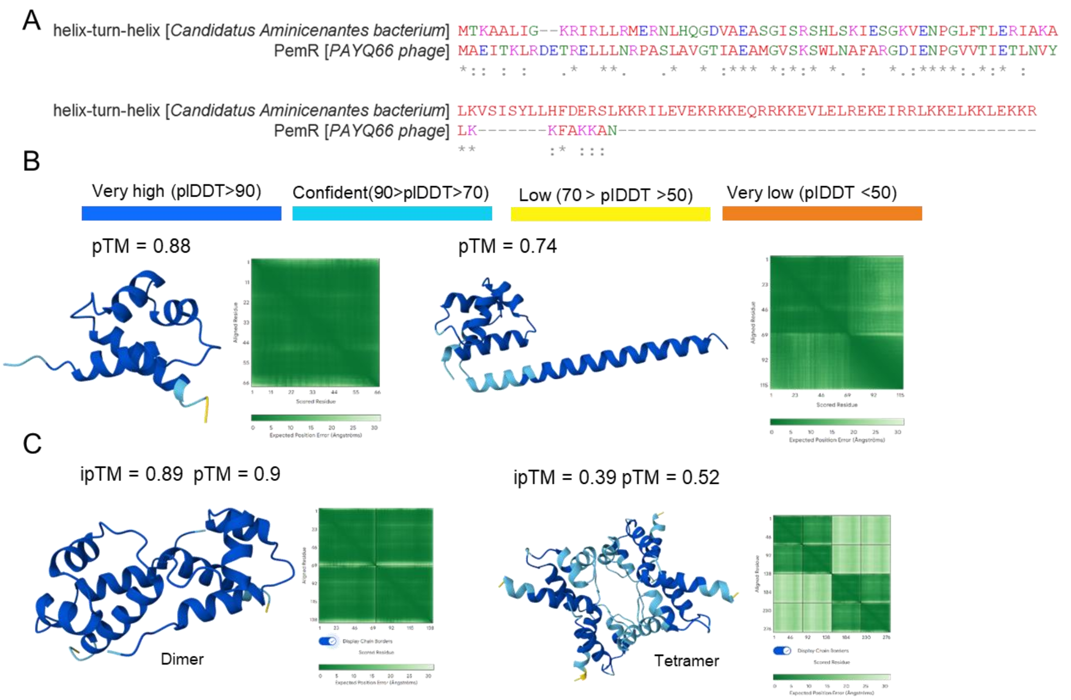

Fig. S4. (A) Amino acid sequence alignment of PemR and helix-turn-helix (HTH) transcriptional regulator from *Candidatus Aminicenantes* bacterium (Sequence ID: UCE40751.1). **(B)** AlphaFold3 structural prediction of PemR and the helix-turn-helix (HTH) transcriptional regulator from *Candidatus Aminicenantes* bacterium. **(C)** AlphaFold3 structural predictions of the dimeric and tetrameric forms of PemR.

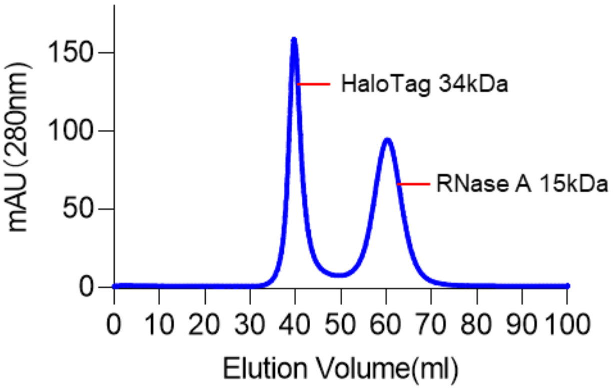

Fig. S5. SEC analysis of Halo Tag (34 kDa) and RNase A (15 kDa). Data shown are representative from two independent experiments.

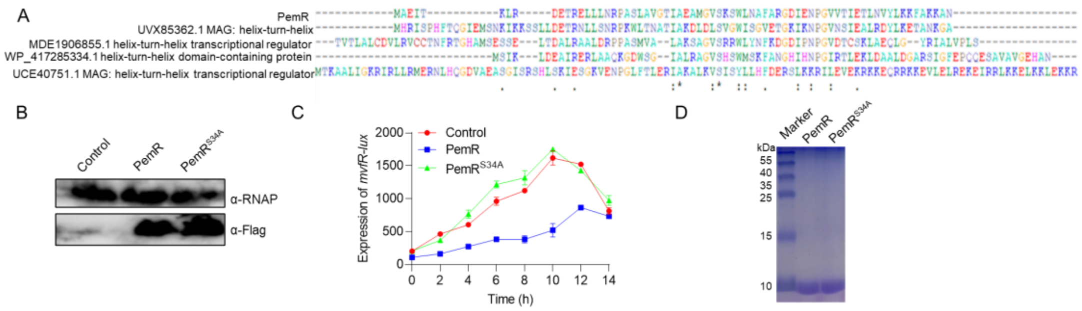

Fig. S6. (A). Multiple sequence alignment of PemR homologs. **(B)** *P. aeruginosa* strains harboring either the empty vector pME6032 (Control), pME6032-*pemR*-Flag (PemR) and pME6032-*pemR*^{S34A}-Flag (PemR^{S34A}) were cultured to an OD_{600} of 1.0. Equal amounts of samples were loaded onto SDS-PAGE gels and detected using anti Flag antibody. α-RNA polymerase (RNAP) served as the loading control. **(C)** PemR^{S34A} lacks the ability to suppress *mvfR* promoter activity, as assessed in PAO1 (Control), PAO1 (PemR), and PAO1 (PemR^{S34A}) strains. **(D)** Gel electrophoretic analysis of purified PemR and the site-directed mutagenesis variants by SDS-PAGE. Gel was stained by Coomassie Blue R-250. (*B* - *D*) Data are representative of three independent replicates. Error bars indicate mean ± standard deviation.

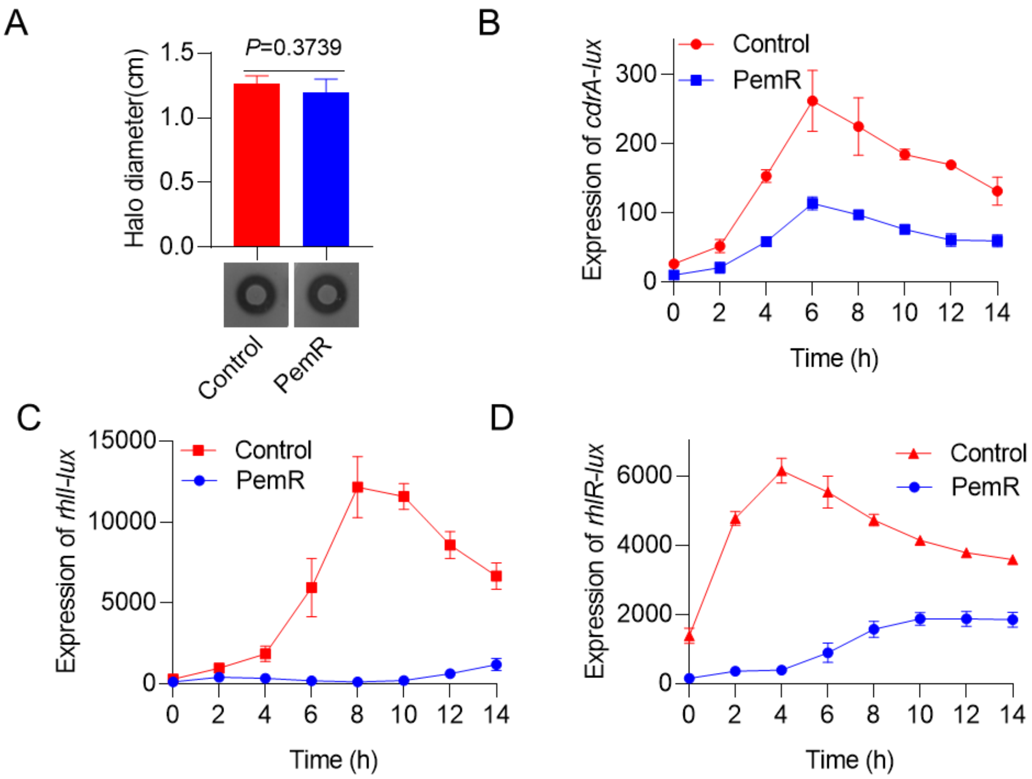

Fig. S7. (A) Protease production by PAO1 (Control) versus PAO1 expressing *pemR* (PemR) on LB agar plate containing milk powder. **(B) PemR inhibits *cdrA* expression.** The transcriptional activity of *cdrA* promoter was analyzed in PAO1 harboring pME6032 (Control) and PAO1 harboring *pemR* (PemR) using reporter gene assay. **(C-D). PemR inhibits *rhlI* and *rhlR* expression.** The transcriptional activity of *rhlI* and *rhlR* promoter was analyzed in PAO1 harboring pME6032 (Control) and PAO1 harboring *pemR* (PemR) using reporter gene assay. (*A - D*) Data shown are representative from three independent experiments. Data are representative of three independent replicates. Error bars indicate mean ± standard deviation.

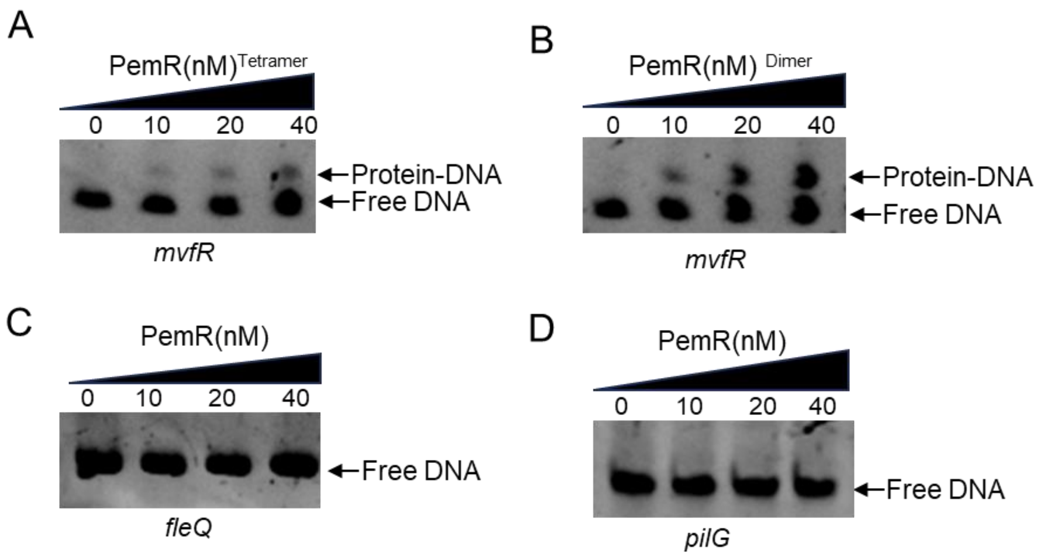

Fig. S8. The EMSA results showed that both the dimer **(A)** and tetramer **(B)** of PemR bind to and shift the *mvfR* promoter. However, PemR does not bind to the promoters of *fleQ* and *pilG* **(C-D)**. Each reaction mixture contained 2.0 ng/μL of PCR products of *mvfR*, *fleQ*, and *pilG*. Protein concentrations are indicated above the lanes.

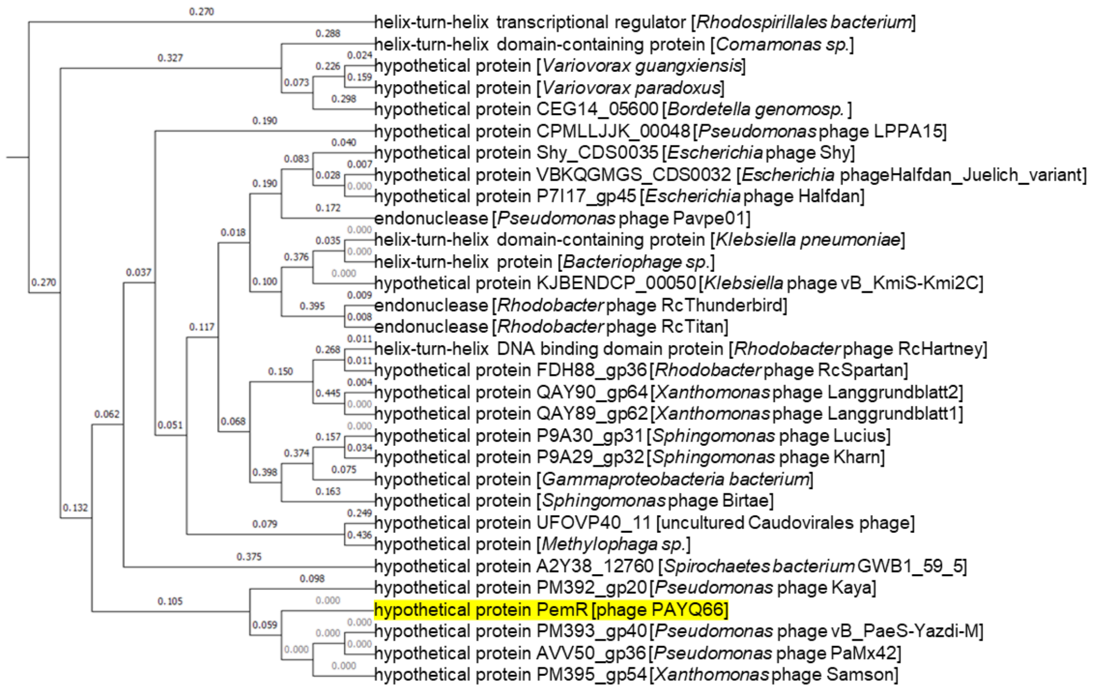

Fig. S9. Phylogenetic relationship for a variety of homologs of PemR, these 31 homologues were acquired with BLASTp against NCBI GenBank as of July 2025.
